# Increased circulating butyrate and ursodeoxycholate during probiotic intervention in humans with type 2 diabetes

**DOI:** 10.1186/s12866-021-02415-8

**Published:** 2022-01-08

**Authors:** Paul J. McMurdie, Magdalena K. Stoeva, Nicholas Justice, Madeleine Nemchek, Christian M. K. Sieber, Surabhi Tyagi, Jessica Gines, Connor T. Skennerton, Michael Souza, Orville Kolterman, John Eid

**Affiliations:** Pendulum Therapeutics, Inc, 933 20th Street, San Francisco, CA 94107 USA

**Keywords:** *Anaerobutyricum hallii*, *Akkermansia muciniphila*, Bile acids, Butyrate, *Clostridium butyricum*, Metabolomics, Short-chain fatty acids, Sulfonylurea, Type 2 diabetes, Ursodeoxycholate

## Abstract

**Background:**

An increasing body of evidence implicates the resident gut microbiota as playing a critical role in type 2 diabetes (T2D) pathogenesis. We previously reported significant improvement in postprandial glucose control in human participants with T2D following 12-week administration of a 5-strain novel probiotic formulation (‘WBF-011’) in a double-blind, randomized, placebo controlled setting (NCT03893422). While the clinical endpoints were encouraging, additional exploratory measurements were needed in order to link the motivating mechanistic hypothesis - increased short-chain fatty acids - with markers of disease.

**Results:**

Here we report targeted and untargeted metabolomic measurements on fasting plasma (*n* = 104) collected at baseline and end of intervention. Butyrate and ursodeoxycholate increased among participants randomized to WBF-011, along with compelling trends between butyrate and glycated haemoglobin (HbA1c). In vitro monoculture experiments demonstrated that the formulation’s *C. butyricum* strain efficiently synthesizes ursodeoxycholate from the primary bile acid chenodeoxycholate during butyrogenic growth. Untargeted metabolomics also revealed coordinated decreases in intermediates of fatty acid oxidation and bilirubin, potential secondary signatures for metabolic improvement. Finally, improvement in HbA1c was limited almost entirely to participants not using sulfonylurea drugs. We show that these drugs can inhibit growth of formulation strains in vitro.

**Conclusion:**

To our knowledge, this is the first description of an increase in circulating butyrate or ursodeoxycholate following a probiotic intervention in humans with T2D, adding support for the possibility of a targeted microbiome-based approach to assist in the management of T2D. The efficient synthesis of UDCA by *C. butyricum* is also likely of interest to investigators of its use as a probiotic in other disease settings. The potential for inhibitory interaction between sulfonylurea drugs and gut microbiota should be considered carefully in the design of future studies.

**Supplementary Information:**

The online version contains supplementary material available at 10.1186/s12866-021-02415-8.

## Background

34.2 million Americans were estimated to have diabetes in 2018 -- over one in ten -- with an estimated annual cost burden of $327 billion and growing, due to an estimated 1.5 million new diagnoses annually [1]. Approximately 90% of total diabetes diagnoses are type 2 (T2D). While genetic factors affect susceptibility, it is increasingly evident that Western lifestyle and diet plays a large role in T2D pathogenesis, as do resident gut microbiota [2, 3]. The therapeutic effects of T2D drugs are at least partially mediated by gut microbes [4–6], including widely-used metformin [7, 8], and many T2D drugs have been shown to alter the gut microbiome [9]. The gut microbiome contributes significantly to predictions of glycemic response [10], and alterations in the gut microbiome induced by lifestyle are mechanistically implicated in aspects of disease progression [11]. Even positive metabolic outcomes from calorie restriction appear to depend on the pre-intervention state of the gut microbiome [12], and certain genera in the healthy human gut are known to be underrepresented in subjects with T2D [11]. Metagenomic surveys have shown that these microbiome alterations often result in a reduction in the capacity or resiliency of short-chain fatty acid production in the gut microbiome, especially butyrate [13].

Dietary fibers, and other dietary oligomers that escape digestion in the upper human gastrointestinal tract, are hydrolyzed and fermented by the microbiota of the lower gut [14], releasing ‘short-chain fatty acids’ (SCFA): 95% of typical SCFA content are the 2C - 4C forms, respectively acetate, propionate, and butyrate [15]. SCFAs are among the most important bacterial metabolites yet identified [2], serving as catabolic substrates for host cell oxidation -- notably butyrate is the primary energy substrate of colonocytes -- as well as direct activation of G-coupled-receptors and inhibition of histone deacetylases [14]. SCFAs can stimulate the proliferation, differentiation and function of Tregs, as well as increase the production of several cytokines (e.g. IL-10) and hence participate in maintaining the balance between pro- and anti-inflammatory immune pathways [16]. SCFAs can enter hepatic or systemic circulation, albeit with molecule-specific transport and fate, where they appear to directly affect metabolism or peripheral tissue function [15]. Robust gut SCFA production has been associated with a reduced risk for certain conditions, including irritable bowel syndrome, inflammatory bowel diseases, cardiovascular disease, and cancer [17] --- particularly colorectal cancer [18–20]. Regarding cardiometabolic health, SCFAs are proposed to beneficially modulate glucose homeostasis and insulin sensitivity via adipose tissue, skeletal muscle, and liver tissue functions. However, there remains a need to fully establish these mechanisms in well-controlled human intervention studies [15].

**Fig. 1:**
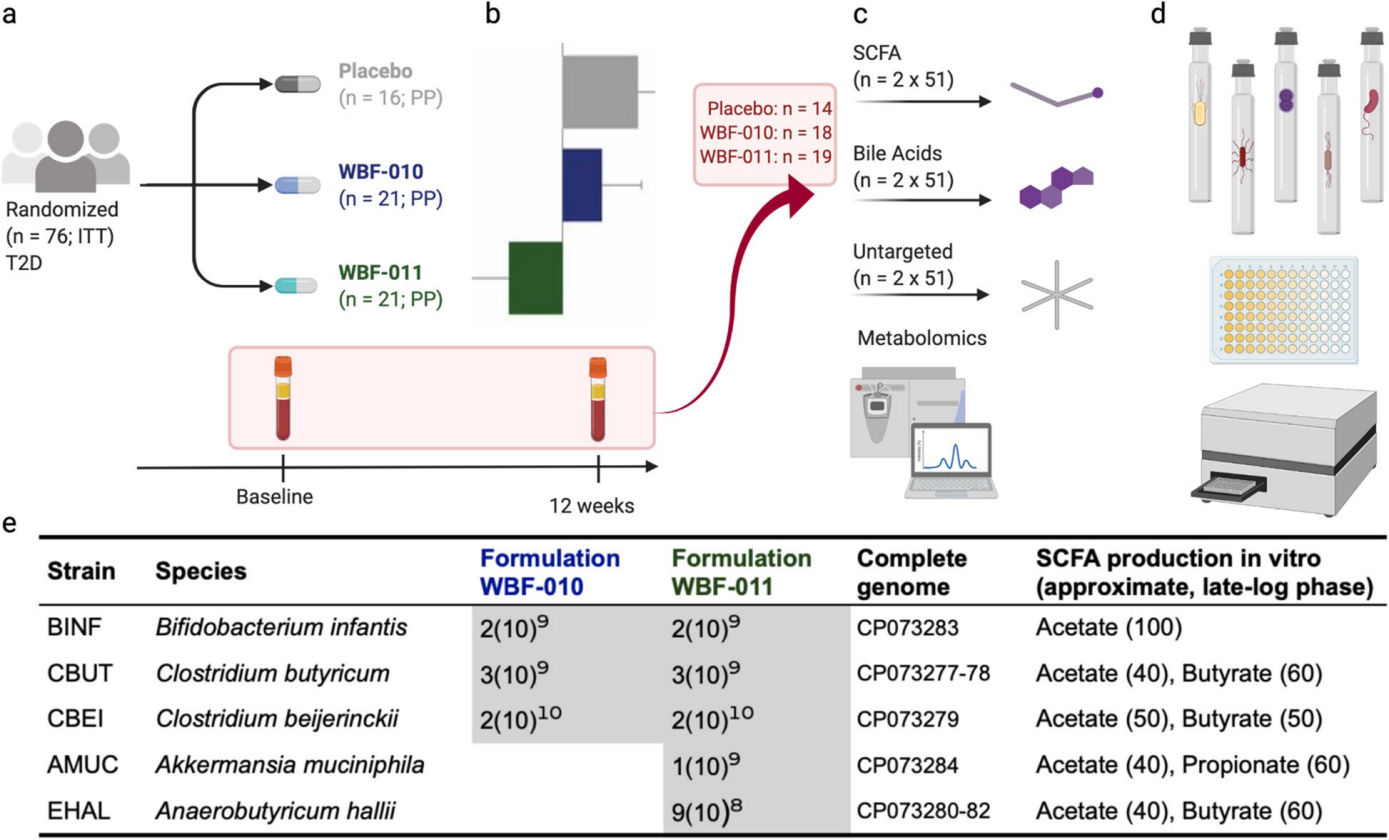
Conceptual outline of study, analyses, and experiments. **a** Clinical study [21] overview including especially the ITT (*n* = 76) and PP (*n* = 58) participant totals, with T2D defined as fasting glucose ≥126 mg/dL or HbA1c ≥6.8%. **b** The fasting plasma specimens were collected during the same visit as the originally-described glucose control endpoints [21]. **c** Of these 58 participants, 51 had successfully collected blood plasma *pairs* suitable for the indicated targeted and untargeted metabolomics analyses. **d** Some of the observations derived from metabolomics were complemented with in vitro monoculture experiments using the formulation strains and additional metabolomics or growth dynamics measurements. **e** Summary of probiotic strains included in the intervention formulations WBF-010 or WBF-011. Identifiers for formulation and strains are the same as in [21]. Entries under each formulation identifier column indicate the approximate viable cell count per daily dose (CFU-equivalent), or absence if blank. Typical in vitro short-chain fatty acid production is indicated for each strain, with approximate ratios at late-log growth phase in VEG (AMUC) or PYG. Complete genome sequencing of each strain was performed, annotated, and deposited in GenBank with the indicated accession numbers

We previously reported initial safety and efficacy for a novel probiotic formulation that induced a significantly improved postprandial glycemic response in participants with type 2 diabetes [21]. The formulation (‘WBF-011’) was hypothesized to produce SCFA in vivo and contained five bacterial strains, each of a different species with varying SCFA production potential [22–25](Fig. 1). Participants randomized to WBF-011 showed an encouraging increase in fecal butyrate concentration that was not observed in participants receiving placebo, although the increase did not reach statistical significance. A known limitation of fecal SCFA measurements is that a variable and overwhelming fraction (90–95%) of luminally-produced SCFA are absorbed prior to excretion in the feces [15, 26], and that circulating SCFA are more predictive for metabolic indicators such as insulin sensitivity, lipolysis, and glucagon-like peptide-1 (GLP-1) concentrations [27].

Some of the aforementioned indicators are also modulated by microbiota-dependent changes to the bile acid pool. Bile acids are critical to dietary absorption of lipids and fat-soluble vitamins, and also regulate numerous host metabolic pathways. Human primary bile acids, cholic acid (CA) and chenodeoxycholic acid (CDCA), themselves implicated in metabolic signaling [28, 29], are synthesized by the liver from cholesterol and conjugated with taurine or glycine by hepatocytes [30] en route to mixing in the gallbladder [31]. Conjugated bile acids are efficiently (~ 95%) reabsorbed in the distal ileum (‘enterohepatic circulation’) [31] following catalysis by gut microbiota of highly-specific epimerization and redox reactions to produce secondary bile acids that alter the composition, solubility, and signaling properties of the bile acid pool [32]. The bile acid receptors, farnesoid X receptor (FXR)] [33] and a G-protein-coupled receptor (TGR5) [34], have elicited considerable interest as targets in metabolic diseases [35], and there is now extensive support for microbiota-dependent modulation of these receptors via modification of bile acids [31].

Here we present analyses of the metabolite content of fasting plasma collected from participants at baseline, and at the end of the randomized intervention. These analyses include targeted measurements of SCFAs and bile acids, as well as untargeted metabolomics. We extend these observations with experiments demonstrating in vitro biosynthesis by formulation strains of metabolites that appeared to increase among participants randomized to WBF-011. We also demonstrate in vitro growth inhibition of some WBF-011 strains exposed to specific sulfonylurea drugs, the use of which appears to have attenuated glycemic improvement during the study.

## Results

### Study overview: formulation, specimens, and data sources

A conceptual outline of the provenance of the data in the present study is shown in Fig. 1. The clinical design of this study was described previously [21] and a concise overview is provided in Fig. 1a-b and in Methods. Extended details into composition of the formulation are shown in Fig. 1e, including the accession numbers for the complete genome sequence of each strain and the typical ratios of major SCFAs produced by each strain after growth in rich media. A total of 76 participants with T2D were randomized as part of the intent-to-treat (ITT) cohort, with 58 successfully completing the study for inclusion in the per-protocol (PP) analysis (Fig. 1a-b). Collection of fasting blood plasma for exploratory analysis occurred at participant visits corresponding to baseline and cessation of intervention (~ 12-weeks after baseline), resulting in 51 participants with successfully collected specimen pairs (and two collection duplicates) for a total of 104 specimens included in the main metabolomics collection. Additional details related to assay types, staging, and measurement vendors are summarized in Table 1. Measurements and replicate totals corresponding to experiments on formulation monocultures are summarized in Table 2.

**Table 1:** Summary of plasma metabolite specimens and measurements

Measurement	Vendor	Source Clinical Sites	No. Participants (per protocol)	No. Plasma Specimens	No. times thawed prior to measurement
Targeted SCFA	Metabolon	Sites 1–5	51	104	1
Untargeted	Metabolon	Sites 1–5	51	104	1
Targeted Bile Acids	MS-Omics	Sites 1–5	51	104	2
Untargeted	MS-Omics	Site 6	10 (4)	20	1

**Table 2:** Summary of in vitro monoculture experiments

Measurement	Vendor	No. of Samples	Strains	Specimen
Untargeted	Metabolon	5 (pilot; 1 per strain)	AMUC, BINF, CBEI, CBUT, EHAL	supernatant, cell pellet
Bile acids	MS-Omics	30 per strain (NIC: 12, Inoc: 18)	AMUC, BINF, CBEI, CBUT, EHAL	supernatant, cell pellet
sulfonylurea sensitivity, growth dynamics	(in-house)	45 per strain per run (3 replicates, 5 titers, 3 SFUs); NIC: 12, DMSO-only: 12, Base medium: 12	AMUC, BINF, CBEI, CBUT, EHAL	N/A

For the untargeted pilot, each strain received separate untargeted measurements of late-log phase supernatant and cell pellet. The uninoculated growth medium was separately measured as well

*NIC* Not-inoculated control, *Inoc* Inoculated, *SFU* Sulfonylurea

### Plasma butyrate increased among the major short-chain fatty acids, correlated with stool butyrate and HbA1c response

**Fig. 2:**
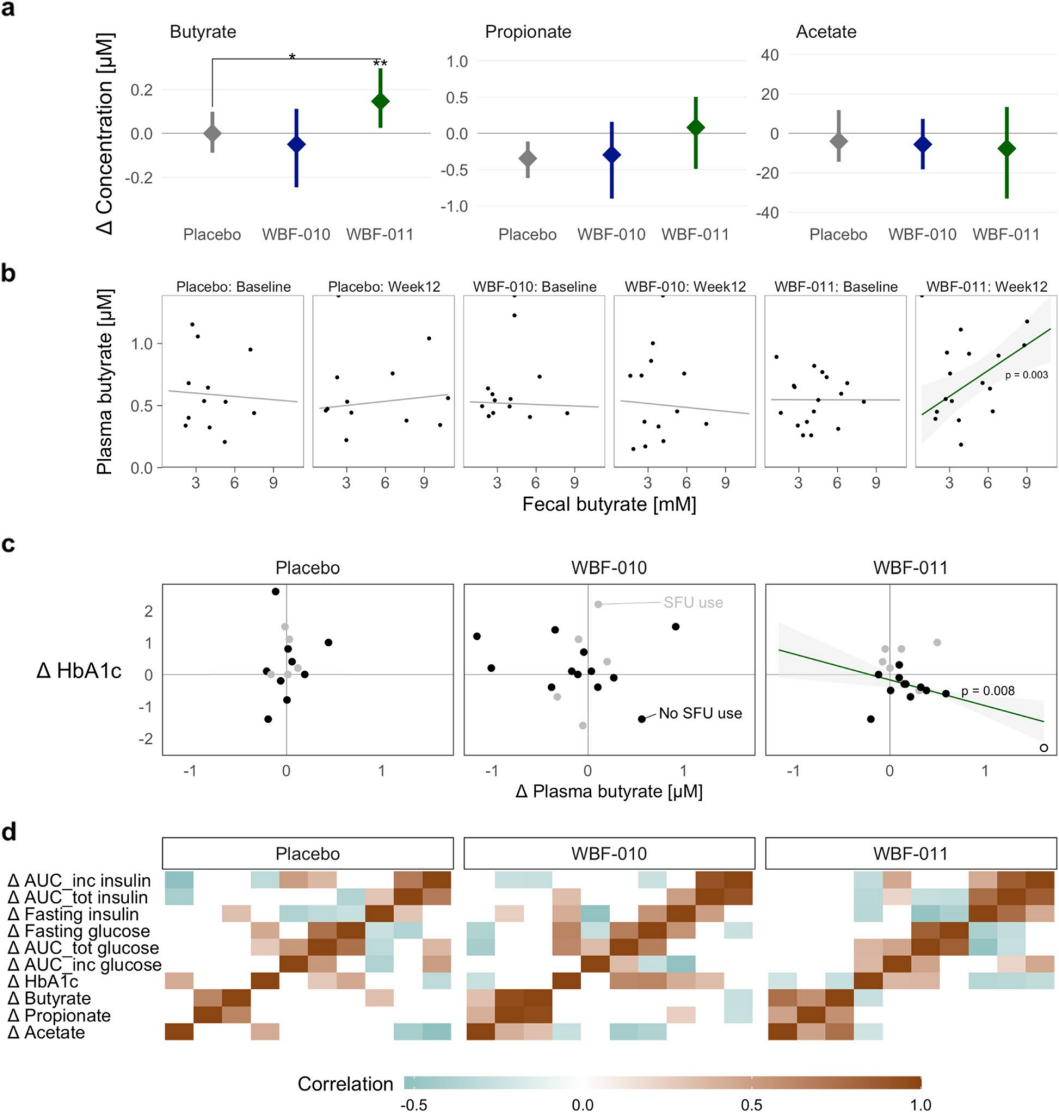
Summary of targeted measurements of short chain fatty acids in human plasma after fasting. **a** Change in fasting circulating (plasma) concentrations [μM] for the three major short chain fatty acids (SCFAs). Line ranges summarize the 95% within-group confidence interval (Wilcoxon Rank Sum test [37]), with nominal significance indicated above the respective group (star) or between-group comparison (bracket with star). Single and double stars indicate the one-sided significance of *p* < 0.05 and *p* = 0.007, respectively. **b** Scatter plot of human plasma butyrate [μM] versus fecal butyrate [mM] corresponding to specimens collected at the same clinical visit. Line indicates the robust linear regression [36], with a green shading and *p*-value shown for the only slope coefficient to reach nominal significance, *p* = 0.003. **c** Scatter plot of the change in participant HbA1c versus their change in plasma butyrate [μM]. Due to the confounding variable of SFU drug use (see below), participants using SFU drugs are highlighted in gray and omitted from the robust linear regressions. In both (b) and (c) the gray ribbon denotes the 95% confidence region for the linear regression. **d** Heatmap of Spearman’s correlation between the participant-wise changes in metabolic measures (top seven rows) and major SCFAs (bottom three rows). Correlations were separately estimated for each study arm. Correlation magnitude and direction is indicated by the provided color shading legend. ‘AUC_inc’ and ‘AUC_tot’ prefixes correspond to the incremental and total area under the curve of the oral meal tolerance test, respectively. For clarity, correlations with nominal *p* > 0.4 are set to zero (white color shading) irrespective of incidental correlation value

Among the three major gut-derived SCFAs (acetate, propionate, butyrate), only butyrate significantly increased (Wilcoxon within-group one-sided increase, *p* = 0.007; WBF-011:placebo between-group one-sided increase, *p* < 0.05), with a median increase in WBF-011 group of 0.15 μM, or 27% increase from baseline after 12 weeks (Fig. 2a). Among the minor SCFAs measured, only valerate (C5) increased significantly relative to placebo (data not shown). The collection of stool and plasma from the same participant and clinical visit allowed for direct correspondence of the SCFAs of these specimen pairs. Among the SCFAs measured, robust linear regression [36] detected a significantly non-zero slope only among the participants in the WBF-011 arm at the end of intervention (Fig. 2b, slope *p* = 0.003). The glycated hemoglobin (HbA1c) value of study participants was also measured at these two clinical visits, and only in WBF-011 group did we detect a significantly negative slope for participant-wise changes in HbA1c and plasma butyrate concentration (Fig. 2c, sulfonylurea non-users, slope *p* = 0.008 with high leverage point omitted, *p* < 10^− 6^ if included; R^2^ = 0.85; Spearman’s *p* < 0.19), consistent with improvement in HbA1c with increasing butyrate. This negative correlation is shown in context with the correlation for other changes in SCFA and metabolic measures in Fig. 2d. That the detected butyrate was derived from the gut is canonical, particularly for plasma collected at the end of a fasting period, and supported by the aforementioned correlation with fecal butyrate of the WBF-011 group at the end of the 12-week intervention (Fig. 2b).

### Plasma ursodeoxycholate increased among the bile acids, potentially due to the activity of *C. butyricum* (CBUT)

**Fig. 3:**
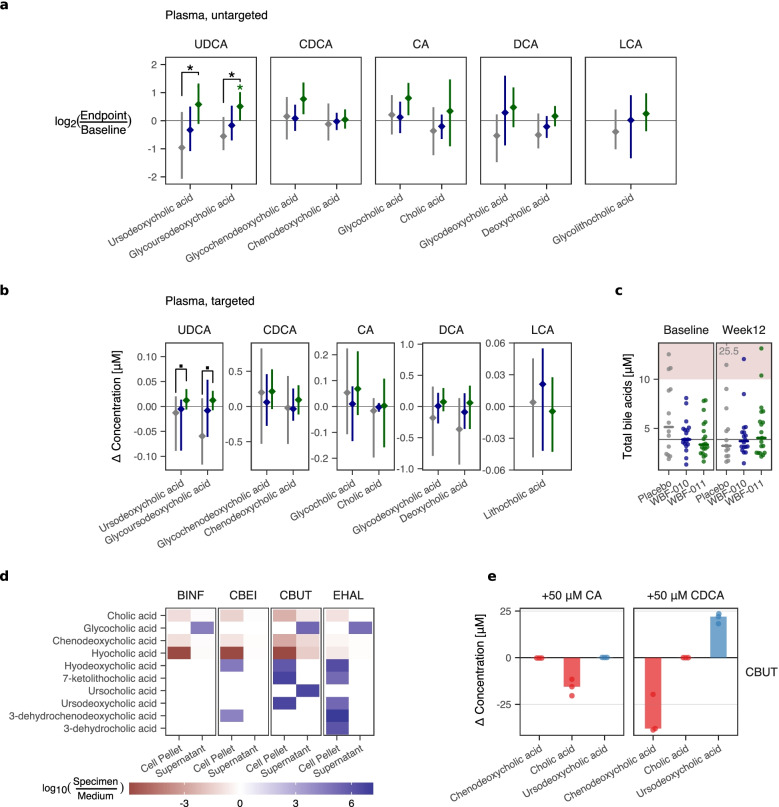
Plasma bile acids and evidence of direct conversion by formulation strains. **a** Changes in selected bile acids from untargeted metabolomics, as log_2_ ratio. Panel label indicates the group corresponding to the unconjugated form. Brackets highlight nominal statistical significance in between-group comparison (UDCA: *p* = 0.016, G-UDCA: *p* = 0.006), while (*) highlights within-group nominal significance (WBF-011, UDCA: *p* = 0.089, G-UDCA: *p* = 0.045). In panels (**a**)-(**c**), gray, blue, and green color represents the Placebo, WBF-010, and WBF-011 groups, respectively. **b** Changes in selected bile acids in targeted data, in micromoles per liter. Ordered as in (**a**) for comparison. Brackets highlight nominal statistical significance in the between-group comparison (UDCA: *p* = 0.075, G-UDCA: *p* = 0.066). **c** Plasma total bile acids. A light red color highlights the beginning of the reference range for (intermediate) hyperbiluremia (10 μM). A gray horizontal line indicates the study grand median at baseline, while a short solid horizontal bar indicates the groupwise median at each timepoint. **d** Summary of bile acids detected via untargeted metabolomics in a pilot in vitro monoculture survey. BINF, CBEI, CBUT and EHAL strains were grown in identical rich medium (PYG) amended with 50 μM each of human primary bile acids, cholic acid and CDCA. Maroon or blue color scale hew corresponds to negative or positive log_10_-ratio values, a decrease or increase in concentration of the specimen relative to the uninoculated medium, respectively. **e** Summary of UDCA synthesis during in vitro monoculture of formulation strains in media amended with 50 μM of the indicated primary bile acid. Only CBUT produced non-negligible UDCA. Red and blue color shading emphasizes detected primary and secondary human bile acids, respectively. Change in concentration is calculated as the average concentration measured in the uninoculated medium subtracted from the volume-weighted concentration of the endpoint cell pellet and supernatant

The results of independent detection and analysis of both untargeted and targeted bile acids for the main collection of 104 plasma samples is shown in Fig. 3a-b, respectively. With only a few exceptions, total bile acids appear to be within the nominal range (2–10 μM) [38] for most participants and timepoints (Fig. 3c). For the untargeted measurements, secondary bile acids rank as the metabolite group (Metabolon “Sub-Pathway”) with the most significant non-zero median difference relative to placebo group (*p* = 0.0056, FDR = 0.12); and among these, glycoursodeoxycholic acid (G-UDCA) and ursodeoxycholic acid (UDCA) had the smallest nominal *p*-values (respectively 0.0062, 0.0162). The targeted measures of bile acids also support a participant-wise increase in concentrations of G-UDCA and UDCA (Fig. 3b, + 0.09 μM, nominal *p* = 0.08).

In order to test the potential for individual strains to modify bile acids directly, we performed in vitro monoculture experiments in growth media amended with primary bile acids. An initial pilot experiment utilizing untargeted culture metabolomics implicated CBUT as the most-likely candidate for UDCA production (Fig. 3d), and this was followed by a targeted/quantitative assay for bile acids assay on fully replicated and controlled in vitro monoculture specimens (Fig. 3e). Only monoculture of CBUT amended with 50 μM of the human primary bile acid, CDCA, resulted in unambiguous synthesis of UDCA, with a conversion of 50–80% after 7 h incubation in rich media (Fig. 3e). As expected, no appreciable UDCA was detected in any conditions amended solely with 50 μM CA. The epimerization of CDCA to UDCA is canonically catalyzed via the action of two enzymes, 7ɑ- and 7β-hydroxysteroid dehydrogenase (7ɑβ-HSDH) [39]. Of the five formulation strains, only the genome of CBUT is predicted to encode 7β-HSDH, and encodes the only 7ɑ-HSDH with UniProt bitscore > 200 (Supplementary Table 1, Supplementary Fig. 1).

### Additional coordinated changes detected via untargeted metabolomics

**Fig. 4:**
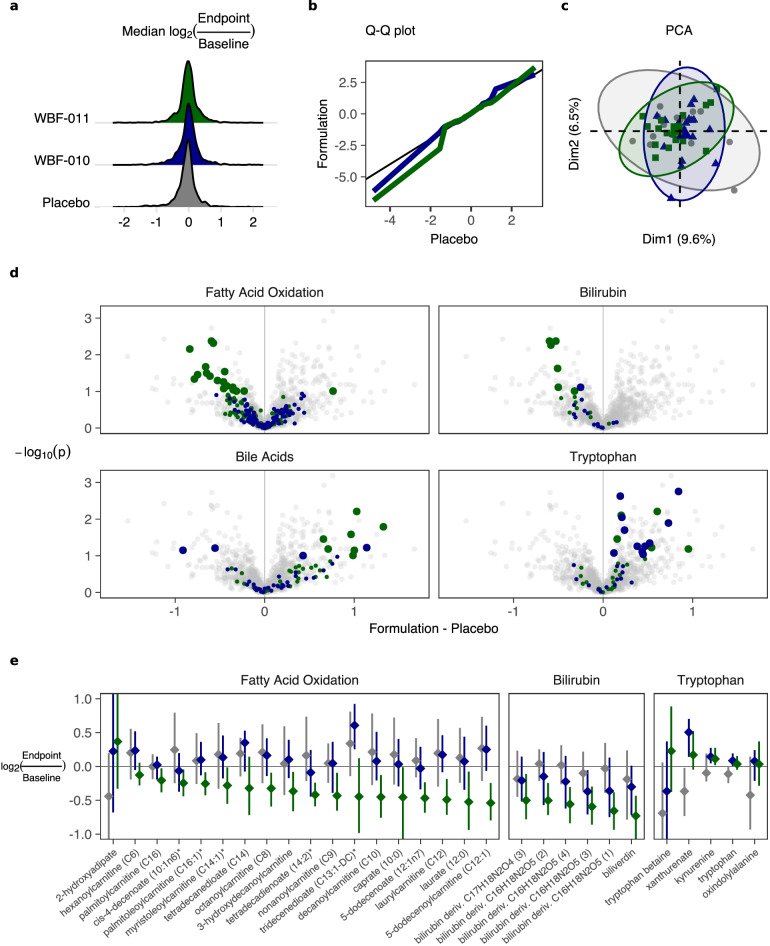
Summary of untargeted metabolomics from fasting plasma. **a** Distribution of the median participant-wise log_2_-ratios of each metabolite. Color palette by study arm is reused in the remaining panels. **b** QQ-plot [40] with 1-percentile steps for each probiotic intervention arm versus placebo. Color shading as in (**a**). **c** Principal Component Analysis (PCA [41]) on the participant-wise log_2_-ratios of each metabolite’s untargeted value. Each point represents a participant, with color shading by arm as in panel (A). Metabolites with low detection prevalence across the cohort were excluded (1156 of 1340 metabolites included). **d** Volcano-plot [42] summarizing the between-group multiple testing (Wilcoxon Rank Sum [37], two-sample, two-sided) of the participant-wise log_2_-ratio of each metabolite. Vertical axis indicates the nominal *p*-value of each metabolite, while the horizontal axis indicates the estimated effect, structured as the Placebo group subtracted from the Formulation group (either WBF-011 or WBF-010). The full volcano scatterplot is repeated as light gray points in separate panels that have an additional layer of green- or blue-shaded points, with each panel emphasizing a different metabolite group of prior interest and apparent coordinated change. **e** Summary of the within-group changes (participant-wise log_2_-ratios) for each study arm by metabolite and metabolite group highlighted in (**d**). Diamond and linerange indicates the group median and Wilcoxon 95% confidence interval, respectively

The initial untargeted survey of 1340 metabolites across 104 plasma specimens supports the null hypothesis that plasma metabolites did not change *en masse* within or between study arms (Fig. 4a-c), and we did not detect notable differences between the arms at baseline. A multivariate summary of metabolite change values (participant-wise log ratio) via standard Principal Component Analysis indicates large overlap between the study arms (Fig. 4c) as well as a diffuse variance explained across many axes.

Multiple testing of within- and between-group changes for individual metabolites revealed a small number of metabolites that do appear to have changed with nominal statistical significance in the WBF-011 arm, and in a few cases these are within groups of interest for plasma metabolites and metabolic diseases (Fig. 4d-e). In particular, many metabolites associated with bilirubin degradation or medium chain acylcarnitines decreased, while a subset of bile acids (described above) and tryptophan intermediates increased. The complete tables of metabolite changes, including those not highlighted here or not associated with an a priori metabolite pathway group, are included in the reproducible data compendium corresponding to Fig. 4. The large number of untargeted metabolite features relative to the number of specimens/participants leads to a challenge to control false discoveries (Type-I error). We endeavored to mitigate this risk by emphasizing groups of metabolites of prior interest that showed encouraging group-wise ranking and FDR. In the case of bile acids including ursodeoxycholate, we also repeated the measurement in a targeted assay with a second metabolomics provider. Aside from Type-I error, poorly annotatable metabolites as well as metabolites not associated with a larger coordinated group tended not to escape standard FDR correction even after independent filtering [43], and this arrangement may have inflated Type-II error due to incomplete knowledge about the identity or pathways of metabolite features, or their relevance.

Butyrate has been shown to induce fatty acid oxidation, lipolysis, and thermogenesis [2], including in the liver [15] and skeletal muscle [44]. This is consistent with our finding of a coordinated decrease in many acylcarnitines and their conjugate fatty acids in the WBF-011 group (‘Fatty Acid Oxidation’ group, Fig. 4d-e). The plasma profile of acylcarnitine esters provides a systemic snapshot of in vivo flux through specific steps of beta-oxidation, due especially to the regulated activity of carnitine acyltransferases [45] that govern the exchange of CoA for carnitine to enable transport across the mitochondrial membrane [46]. Adults that are obese or have T2D often exhibit elevated levels of acylcarnitines and free fatty acids in plasma [47–49], along with their conjugate fatty acids, derivatives of branched-chain and aromatic amino acids, and β-hydroxybutyrylcarnitine [50, 51]. We did not detect significant changes in the respective ensembles of branched-chain or aromatic amino acid plasma metabolites.

### Sulfonylurea drug use stratifies glucose control response

Three distinct sulfonylurea (SFU) drugs - glipizide, glimepiride, and glyburide - were detected in the plasma of some members of the study cohort. This includes a total of eight participants (placebo: 1, WBF-010: 3, WBF-011: 4) that had unambiguous levels of SFU drug at one or both collection events despite having no reported usage. The detection of SFU drugs in plasma through untargeted metabolomics appears to be reliable. The detection of SFU drugs was unambiguous for SFU presence (ratio of signal to limit-of-detection > 10 in the mildest case, > 10^3^ in most cases); there was perfect agreement (57/57) on SFU presence/absence between timepoints from the same participant; a majority of participants (15 / 19) showed agreement between timepoints regarding *which* SFU drug was detected (same molecule); and a majority of participants (*recall*, 11 / 13) recording SFU use had positive detection in their plasma. All SFU drugs are taken orally and absorbed in the intestines, with plasma half-lives (glipizide: 2–4 h, glimepiride: 5–8 h, glyburide: 4–10 h) and fecal elimination percentages (glipizide: 10%, glimepiride: 40%, glyburide: 50%) that vary by drug [52, 53]. These timescales and the previously-described consistency between timepoints indicates that the detection of SFU drugs in plasma was due to recent and likely-ongoing use.

**Fig. 5:**
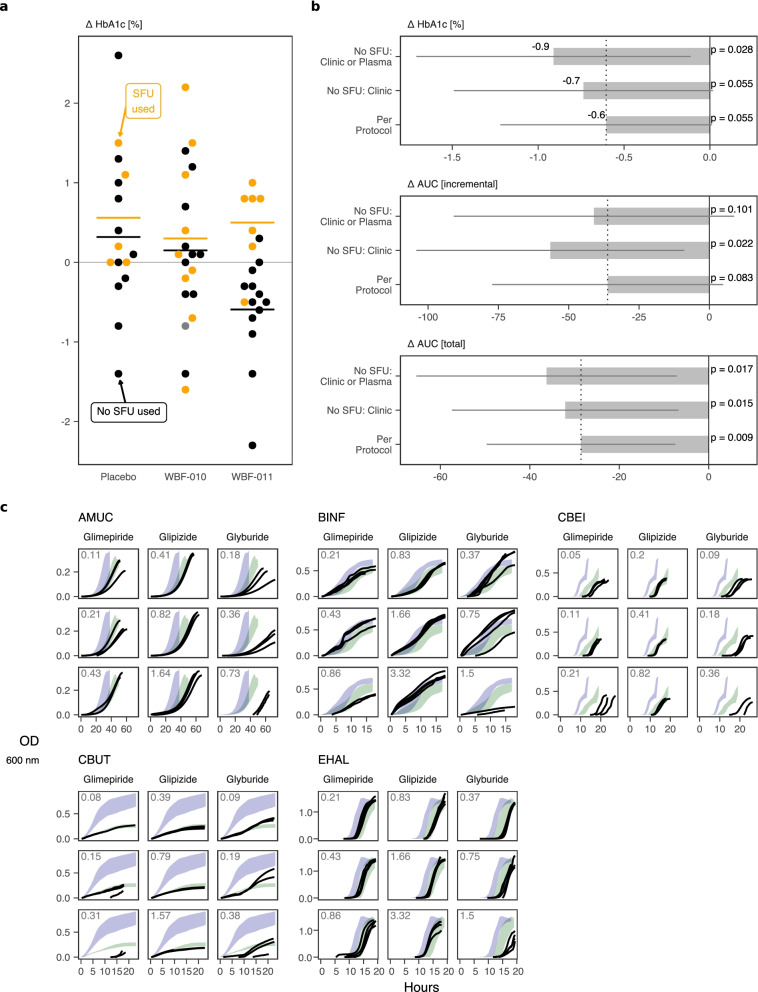
Sulfonylurea drugs: use stratifies glucose control endpoints, and some formulation strains are inhibited by SFUs in a drug-specific pattern. **a** Participant change in HbA1c versus study arm, with metabolomics-enhanced SFU drug usage status indicated by color shading. Gold and black color respectively indicate participants that are believed to have used, or not-used, SFU drug during the study as determined from the clinical record, direct observation of SFU in blood plasma, or both. Cross-bar indicates the value of each sub-group mean. **b** Summary of the re-estimation of glucose control endpoint statistics in the comparison of WBF-011 group (the five strain formulation) versus Placebo group (shown as WBF-011 - Placebo). ‘Per protocol’ refers to the cohort that successfully completed the study, as described previously [21]. ‘No SFU: Clinic’ refers to the results of between-group comparison among participants not known to use SFU according to the clinical record. This is equivalent to the confounding effect described in the original study. ‘No SFU: Clinic or Plasma’ is the same analysis, but with additional participants omitted where SFU was detected in metabolomics. **c** Representative growth curves (OD 600 nm versus time in hours) for formulation strains with or without the presence of the indicated SFU drug in mVEG (AMUC), mPYG (CBEI, CBUT), or PYG (BINF, EHAL). Panel rows from top to bottom represent a 2-fold increasing concentration of the indicated SFU, with the millimolar concentration labeled in the top-left corner. Each black curve is a separate replicate inoculated at the same time as other curves for that strain, including positive controls. Green and blue regions indicate the range occupied by no-SFU positive controls in the same medium, with or without the final volumetric fraction of DMSO (3% for CBUT, 2% for all others), respectively. OD 600 nm values have been spline-smoothed and baseline-subtracted. To improve interpretability, each curve has been filtered to display just the period of continuous monotonic increase (growth)

The additional participants determined through metabolomics to be using sulfonylurea drugs are overrepresented in the glycemically nonresponsive group, strengthening the post hoc endpoint stratification (Fig. 5a-b) [21]. Now, six of the seven participants with ΔHbA1c > 0 (worse) in the WBF-011 group had detectable SFU drug, compared with three in the original clinical record. Placebo and WBF-010 groups showed only mild differences in ΔHbA1c between SFU users and non-users (Fig. 5a), such that the corresponding post hoc statistics for all glucose control endpoints improved for WBF-011 upon omission of SFU users; including a mean ΔHbA1c relative to placebo that improves from − 0.6 originally to − 0.9, with improved nominal significance (Fig. 5b).

Given SFU intestinal absorption and non-trivial fecal excretion [52], we could not exclude a priori that direct inhibitory interaction may occur between a consumed SFU drug and the live strains of an orally administered probiotic. In order to test whether SFU could directly inhibit the formulation strains, each was grown in monoculture in dilute rich media with or without the presence of each of the three detected SFUs. The SFU concentration was titrated in replicated conditions that spanned what might be expected in vivo according to typical daily dosing, average large intestinal volume, and solubility limits. Unambiguous concentration-dependent growth inhibition was observed (yield decrease, lag increase) for certain strains and SFUs (Fig. 5c). Glyburide and glimepiride exhibited the largest inhibitory effect overall, with patterns that were strain-specific. Importantly, glipizide was used by 5 of 7 participants using SFUs in the WBF-011 group and exhibited a substantially muted inhibitory effect under these conditions (Fig. 5c), consistent with a limited microbiome impact by glipizide reported in another study [6], complicating interpretation and suggesting additional or separate mechanisms may be required to explain the observed effect stratification.

## Discussion

While there is compelling human and animal data supporting the role of gut microbiota in metabolic health and disease, there is limited probiotic interventional data in humans with diabetes that shows an improvement in clinical endpoints while also demonstrating significant changes in the hypothesized metabolite mediators of the response. While human data has not been available, multiple rodent studies have demonstrated that increased butyrate production by fermentation of dietary fiber within the gut microbiome leads to increased secretion of GLP-1 by L-cells within the mucosal lining of the intestine [54]. GLP-1 is recognized to play a central role in glucose homeostasis through augmentation of glucose-dependent insulin secretion, suppression of inappropriate glucagon secretion, and reduction of hepatic glucose production. GLP-1 also participates in appetite suppression via direct CNS effects initiated in the hindbrain in conjunction with slowing of gastric emptying. These desired attributes have led to the present widespread use of GLP-1 receptor agonists in the clinical management of type 2 diabetes [55].

In the present study, we performed SCFA, bile acid, and untargeted metabolomics analyses of circulating metabolites from the plasma of human participants with T2D collected before and after a randomized, double-blind placebo controlled probiotic intervention that resulted in improved glucose control endpoints [21]. The major findings of our analysis were: (1) circulating butyrate significantly increased in the participants administered the 5-strain probiotic formulation, consistent with the original motivating hypothesis of the study; (2) circulating (G-)UDCA also significantly increased in these participants, representing a potential complementary or synergistic avenue for affecting the observed improvements; (3) one of the formulation strains, CBUT, performs the epimerization of CDCA to UDCA with a high conversion frequency, a somewhat rare capability that was not widely reported for this species previously; (4) untargeted metabolomics identified additional usage of SFU drugs by participants than previously understood, revealing a striking stratification of SFU-use and HbA1c non-response in the WBF-011 group only; (5) two of the three SFU drugs caused unambiguous growth inhibition of the probiotic strains during in vitro monoculture, representing a poorly explored potential side-effect for a drug class that is administered widely and is incompletely absorbed in the intestines.

Data on the microbiome impact of sulfonylurea drug class is notably lacking [56]. Anti-diabetic SFUs were originally discovered in 1942 during research on bacteriostatic sulfonamides [57], with demonstrated bacteriostatic properties via inhibition of acetohydroxyacid synthase (AHAS, EC 2.2.1.6) critical to synthesis of branched chain amino acids (BCAA) [58, 59]. Preliminary attempts to ameliorate growth inhibition via amendment of BCAAs to the media --- as would be expected if each SFU effect was exclusively due to BCAA limitation derived from AHAS inhibition --- were unsuccessful (data not shown). However, the unambiguous in vitro growth inhibition by glyburide and glimepiride supports the potential that this common T2D drug class may inhibit these probiotic strains in vivo, thereby contributing to the apparent non-response of participants taking SFU drugs in this study, and suggesting that future studies utilizing probiotic strains should consider in advance the SFU usage status of recruited participants. The observed effect of SFU-use on HbA1c can be explained by: (1) SFUs directly inhibit activity or growth of one or more of the WBF-011 formulation strains, (2) SFUs impact host glycemic control (e.g. constitutive enhancement of insulin secretion) in a way that abrogates the effect observed among the no-SFU participants randomized to WBF-011, (3) SFU usage correlates with a clinical stage of T2D that is more advanced, limiting capacity for improvement during the study. The present study design cannot distinguish between these explanations, nor rule out that they are contributing simultaneously.

The magnitude of the increase in plasma butyrate (+ 27%) and improvement in HbA1c [%] (− 0.6) appear to be notable among microbiome-oriented (prebiotic, probiotic) interventions in human participants with T2D. Independent meta-analyses of probiotic [60], dietary [61], and fiber [62] interventions in human participants with T2D have described mean improvements in HbA1c [%] of − 0.14, − 0.21, and − 0.18, respectively, with a 95% confidence interval *lower* bound ≥ − 0.34 [60]. Of these meta-analyses, only (ref [62] reported on SCFA changes, finding a significant increase in total *fecal* SCFAs, but not fecal butyrate, and no circulating SCFA changes reported. In a recently reported isocaloric intervention in human participants with prehypertension or stage 1 hypertension, Mueller et al. show a significant increase in circulating acetate (+ 10%) -- as well as butyrate (+ 5%) in one of the three high-fiber diets -- along with significant improvements in fasting blood glucose, but not HbA1c [63]. A recent study of 24 participants with metabolic syndrome administered a single-strain probiotic of *Anaerobutyricum soehngenii*, with limited evidence of improvements in insulin sensitivity and no detected increases in fecal SCFAs (circulating SCFAs were not measured) [64].

The plasma SCFA concentration ranges reported here were largely within commonly reported levels. However, it may be informative for future studies to note that butyrate was relatively low at baseline for all groups (median = 0.5 μM, std. dev. = 0.3 μM), compared with a typical range of 1–12 μM [15]. Although much smaller than typical fecal concentrations (1–20 mM) [15], plasma butyrate is only the trace *spillover* from a highly efficient colonic and hepatic absorption, with ^13^C stable isotope experiments indicating only about 2% of butyrate flux ultimately arrives into circulation [65]. Therefore, the observed butyrate median relative change of + 27% is likely to be more useful than + 0.15 μM for intuition and cross-study comparison. The highly leveraged implication of changes to circulating butyrate concentration likely explains its more robust predictive value for markers of metabolic syndrome [27]. Compared to the present study, larger baseline levels (~ 2 μM) and interventional increases (+ 1 μM) in fasting plasma butyrate were observed in healthy young adults receiving a night-time administration of arabinoxylan oligosaccharides -- and SCFA increases correlated with improvements in glycemic response [66]. The present study does not address whether additional butyrate increases would have been possible with a companion prebiotic supplement like arabinoxylan oligosaccharides.

Analogous to butyrate, circulating concentrations of bile acids are typically only a small fractional ‘spillover’ from enterohepatic circulation, complicating interpretation but also suggesting that the observed increases of (G-)UDCA in plasma represent much larger localized increases in intestinal concentration and flux. Although there appears to be a possible trend for a within-group increase in (G-)CDCA, we found only limited evidence for increases in the substrates from which UDCA is typically derived (CDCA, lithocholic acid, all between-group *p* > 0.19), suggesting the increase is driven by increased activity of UDCA-generating microbes. Confirmation via in vitro monoculture that CBUT efficiently converts CDCA to UDCA suggests that at least some of the observed increases in UDCA could be explained by direct activity of the WBF-011 formulation in vivo. Several human gut bacteria (including *Clostridia*) have been described to convert CDCA to UDCA [39], but to our knowledge, there is only limited prior description of *C. butyricum*’s ability to catalyze this reaction [67] despite the use of *C. butyricum* as a probiotic species in overlapping indications with UDCA [68–70].

The observed increases in circulating butyrate and UDCA following administration of a 5-strain probiotic that included a strain of *Akkermansia* may represent potential synergistic mechanisms. Recent studies have described an *Akkermansia*-enhancing activity of UDCA or its conjugates [71, 72]. UDCA is also unique among major human bile acid constituents in that it antagonizes FXR in intestinal L cells [31, 73], while the human primary bile acids are FXR agonists that inhibit synthesis of GLP-1 in these same cells [31]. Butyrate stimulates secretion of GLP-1 [74–76], a major mechanism for GLP-1-mediated augmentation of the postprandial insulin response [77] by L cells of the small intestine [78]. Butyrate may also act synergistically, alongside FXR inhibition by UDCA, to further enhance GLP-1 secretion [79]. A recent study [80] describes induction of GLP-1 secretion via *Akkermansia muciniphila* ATCC-BAA-835 secreted protein, “P9”, that is 100% identical to the P9 encoded in the genome of strain AMUC (CP073284.1 locus KDJ95_08250), implying another potential avenue for enhancement of GLP-1 secretion. It is tempting to speculate that a postprandial increase in concentrations of butyrate and UDCA in the proximal gut --- with a concomitant decrease in FXR-*activating* T-(G-)CDCA and formulation- or UDCA-augmented *Akkermansia* and its secreted protein P9 --- results in a synergistic increase in postprandial GLP-1 secretion with benefits for glucose homeostasis.

## Conclusion

Taken together, these observations provide additional support for the plausibility of a probiotic intervention to affect improvements in glucose control and associated improvements in metabolic health, while contributing to a broader metabolic context for guiding future confirmatory and mechanistic studies. These observations are consistent with an emerging understanding that gut-derived microbial metabolites can have direct causal relationships with human health [81], and that these relationships can be negatively altered in T2D through lifestyle-induced loss of ‘key but not keystone’ microbial taxa [82], ultimately requiring reintroduction to regain function [83]. Future studies seem justified for confirmation and to assess whether additional increases in butyrate or UDCA are possible and would enhance improvements in glycemic control in individuals with T2D. The most critical considerations are a larger cohort of participants with T2D treated with metformin monotherapy; a longer duration of intervention; the inclusion of a companion prebiotic or additional butyrate-producing taxa; the direct measurement of GLP-1; and the collection of additional blood plasma specimens, especially during the oral meal tolerance tests to allow for identification of transient phenomena that manifest only during the postprandial period.

## Methods

### Blood plasma sampling and design

The clinical design of this study was described previously [21]. Briefly, adult human volunteer participants were considered eligible if they were diagnosed with T2D --- fasting glucose ≥126 mg/dL or HbA1c of ≥6.8% --- with a body mass index between 25 and 45 kg/m^2^, and treated with diet and exercise alone, or in combination with metformin with or without a sulfonylurea. Recruited participants were randomized to administration of one of three study formulations present in otherwise identical capsules, one of which contained only known inactive ingredients (placebo). Collection of fasting blood plasma for exploratory analysis occurred at a participant’s baseline visit (prior to capsule administration) and at the designated visit completing the intervention period (the end of capsule administration) approximately 12-weeks after baseline. A total of 58 participants successfully completed the protocol and were included in final analyses. Each blood plasma specimen was derived from 10 mL of collected blood, which was allowed to clot for 30 min, and then spun at 1000–1300 xg for 20 min to collect the serum. Samples were maintained frozen (− 20 °C) during on-premises storage at the clinic, then shipped on dry ice where they were stored at our facility at − 80 °C prior to measurement at Metabolon, Inc. (Research Triangle Park, NC, USA) or MS-Omics (Vedbæk, Denmark). The blood plasma specimens from all participants at one of the six study sites (four participants total) were excluded from the original metabolomics measurements due to a critical omission in dating the collected specimens. The fasting blood plasma from the remaining 51 study participants collected at the beginning and end of intervention, as well as two additional collection replicates (104 total specimens), were included in the main survey collection on which metabolite measurements were conducted (Table 1). All 104 plasma specimens were included in a targeted SCFA assay and an untargeted (metabolomics) assay conducted by Metabolon, as well as a targeted bile acids assay conducted by MS-Omics.

### Targeted SCFA measurement, metabolon

Targeted measurement survey of SCFA in the human plasma samples was accomplished via Ultrahigh Performance Liquid Chromatography-Tandem Mass Spectroscopy (Metabolon Method TAM148: “LC-MS/MS Method for the Quantitation of Short Chain Fatty Acid (C2 to C6) in Human Plasma and Serum”). The method utilizes a Waters ACQUITY ultra-performance liquid chromatography (UPLC) and a Thermo Scientific Q-Exactive high resolution/accurate mass spectrometer interfaced with a heated electrospray ionization (HESI-II) source and Orbitrap mass analyzer operated at 35,000 mass resolution. The following eight short or branched chain fatty acids were quantitated: acetic acid (C2), propionic acid (C3), isobutyric acid (C4, branched), butyric acid (C4), 2-methyl-butyric acid (C5, branched), isovaleric acid (C5, branched), valeric acid (C5), and hexanoic acid (C6). The human plasma samples were spiked with stable labelled internal standards, homogenized, and subjected to protein precipitation with an organic solvent. Following centrifugation, an aliquot of the supernatant was derivatized, and the reaction mixture then injected onto an Agilent 1290/AB Sciex QTrap 5500 LC MS/MS system equipped with a C18 reversed phase UHPLC column. The mass spectrometer was operated in negative mode using electrospray ionization. Quantitation was accomplished by adjusting the peak area of the individual analyte product ions with the peak area of the product ions of the corresponding internal standards, and via a weighted linear least squares regression analysis generated from fortified calibration standards prepared immediately prior to each run. Accuracy was evaluated using the corresponding QC replicates. LC-MS/MS raw data were collected and processed using SCIEX OS-MQ software v1.7.

### Untargeted metabolomics measurement, metabolon

Untargeted metabolomics was generated by UHPLC-MS/MS, as above. Several recovery standards were added prior to the first step in the extraction process for quality control and standardization. Samples were then extracted with methanol under vigorous shaking for 2 min (Glen Mills GenoGrinder 2000) to precipitate protein and dissociate small molecules bound to protein or trapped in the precipitated protein matrix, followed by centrifugation to recover chemically diverse metabolites. Extracts are placed briefly on a TurboVap® (Zymark) to remove the organic solvent, and then stored overnight under nitrogen. The resulting extract was divided into the following five fractions: two for analysis by two separate reverse phase (RP)/UPLC-MS/MS methods using positive ion mode electrospray ionization (ESI), one for analysis by RP/UPLC-MS/MS using negative ion mode ESI, one for analysis by HILIC/UPLC-MS/MS using negative ion mode ESI, and one reserved for backup. The untargeted metabolomics platform identified 1340 metabolites in these same specimens with semi-quantitative precision that allows for relative comparison between samples, but not direct measures of concentration and with limited comparison between different metabolites. Note that untargeted metabolomics and targeted SCFA assays were conducted concurrently from separate aliquots during the same thawing event, such that the plasma samples were only thawed once at Metabolon facilities and otherwise maintained at cryogenic temperatures while in storage or in transit.

### Targeted bile acids measurement, MS-omics

A targeted bile acids assay was performed by MS-Omics on the remaining human plasma (twice-thawed prior to measurement) and in vitro culture specimens, for the quantitation of 18 of the most commonly encountered bile acids. Briefly, sample analysis was carried out using a Thermo Scientific Vanquish LC coupled to Thermo Q Exactive HF MS via electrospray ionization, performed in negative ionization mode. The chromatographic separation of bile acids was carried out on a Waters Acquity HSS T3 1.8 μm 2.1 × 150 mm (Waters). The column was thermostated at 30 °C, with the mobile phases consisting of (A) ammonium acetate [10 mM], and (B) methanol:acetonitrile [1:1, v/v]. Bile acids were eluted by increasing B in A from 45 to 100% for 16 min, and a flow rate of 0.3 per minute. Peak areas were extracted using Tracefinder 4.1 (Thermo Scientific). Identification of compounds were based on mass and retention time of standards. An internal standard and a mixed pooled sample were analyzed at regular intervals for quality control.

### Untargeted metabolomics measurement, MS-omics

An additional 20 plasma specimens from a sixth study site (8 specimens from per-protocol cohort, 12 additional specimens from participants that did not successfully complete the study) were collected (Table 1), but with a study event that was not adequately recorded to distinguish a participant’s baseline and endpoint specimens. Untargeted metabolomics survey was performed on these samples by MS-Omics, with the primary goal of identifying whether sulfonylurea use could be detected or confirmed irrespective of study event. Briefly, the assay was carried out using a Thermo Scientific Vanquish LC coupled to Thermo Q Exactive HF MS with electrospray ionization interface. Analysis was performed in negative and positive ionization mode. The UPLC was performed using a slightly modified version of the protocol described in [84]. Peak areas were extracted using Compound Discoverer 3.1 (Thermo Scientific). In general the annotation of features as identifiable molecules occurred at four levels of decreasing qualitative confidence. The detection of sulfonylurea corresponded to ‘Level 2b’, identification by both accurate mass (with an accepted deviation of 3 ppm) and MS/MS spectra.

### In vitro bacterial probiotic strains monoculture

In vitro monoculture experiments were conducted on formulation strains for metabolomics survey, determination of bile acid conversion, and evaluation of sensitivity to sulfonylurea drugs.

An initial pilot, untargeted metabolomics survey was conducted with each strain (*n* = 1 per strain). Anaerobic growth was initiated on solid Peptone, Yeast Extract, Glucose medium (PYG, Anaerobe Systems ref. AS-8228) at 37 °C for 24-96 h in order to obtain single colonies. Single colonies were used to inoculate single, anoxic, 8 mL liquid hungate tubes containing PYG (Anaerobe Systems ref. AS-822) supplemented with 50 μM chenodeoxycholic acid (CDCA, VWR, ref. 10,003–180) and 50 μM cholic acid (CA, VWR, ref. AAA11257–14) from ethanol stocks (< 1% ethanol final concentration). AMUC was grown in vegetable-based medium (VEG, 5.08 g/L NaCl, 0.4 g/L NaHCO_3_, 0.04 g/L KH_2_PO_4_, 2.04 g/L K_2_HPO_4_, 0.02 g/L MgSO_4_·7H_2_O, 0.02 g/L CaCl_2_, 2.5 g/L Na_2_HPO_4_, 0.5 g/L cysteine-HCl, 2 g/L dextrose, 2 g/L N-Acetylglucosamine, 7.5 g/L HiVeg™ Special Infusion, 10 g/L HiVeg™ Extract No. 2, 10 g/L HiVeg™ Peptone No. 3) without the amendment of bile acids due to preliminary data indicating this results in a notable growth lag and AMUC had limited genomic prediction for bile acids modification (Supplementary Fig. 1). Cultures were grown at 37 °C, OD 600 nm was monitored and sampling was performed at mid to late log phase (OD range 2–5, depending on the strain). Each culture was centrifuged at 4 °C for 5 min at 5000 x g, the supernatant was filtered through 0.2 um pore size, and filtrate stored at − 80 °C. Cell pellets were washed twice in phosphate-buffered saline (PBS, ThermoFisher Scientific, 20,012,050), split into duplicate aliquots and stored at -80 °C. Blank (uninoculated) media controls were prepared alongside cultures, as per the above protocol. All supernatants and cell pellets were shipped on dry ice to Metabolon for sample extraction and untargeted metabolomics analysis (as described above).

Culturing and sample preparation for analysis of bile acid transformation by WBF-011 strains was accomplished with growth conditions as above, with several modifications. Single colonies of CBUT, CBEI and EHAL were used to inoculate anoxic PYG medium, cultured to mid-log, and subsequently used to inoculate triplicate tubes, at an initial OD 600 nm of ~ 0.05–0.1, for each of the two conditions: amended with 50 μM CA, amended with 50 μM CDCA. No-inoculum controls (blank media) were processed in triplicate, in parallel, for each condition. Initial samples (t_0_) and final samples (t_f_) were collected as above, without the washing of cell pellets in PBS. Wet weight for each cell pellet was recorded. Complete summary of design with replication and controls provided in Supplementary Table 2.

Growth sensitivity to SFU was performed in 96-well plates with 200 μL well volume, incubated within an anoxic chamber (Coy Laboratory Products). Each strain was separately cultivated in anoxic medium. CBEI and CBUT were cultivated in modified dilute PYG medium (mPYG, 0.08 g/L NaCl, 0.4 g/L NaHCO_3_, 0.04 g/L KH_2_PO_4_, 0.02 g/L MgSO_4_·7H_2_O, 2.04 g/L K_2_HPO_4_, 0.02 g/L CaCl_2_, 0.5 g/L cysteine-HCl, 5 g/L dextrose, 1 g/L yeast extract, 0.5 g/L HiVeg™ Peptone No. 1). AMUC was cultured in anoxic dilute vegetable-based medium (mVEG, 5.08 g/L NaCl, 0.4 g/L NaHCO_3_, 0.04 g/L KH_2_PO_4_, 2.04 g/L K_2_HPO_4_, 0.02 g/L MgSO_4_·7H_2_O, 0.02 g/L CaCl_2_, 2.5 g/L Na_2_HPO_4_, 0.5 g/L cysteine-HCl, 2 g/L dextrose, 4 g/L N-Acetylglucosamine, 1.19 g/L L-threonine, 1 g/L yeast extract, 1 g/L HiVeg™ Acid Hydrolysate). EHAL and BINF were cultivated in PYG (Anaerobe Systems ref. AS-8228). In ‘+BCAA’ conditions, the entire design of the SFU sensitivity was replicated, and branched chain amino acids (L-valine, L-leucine and L-isoleucine, Millipore Sigma, V0513, L8000 and I2752) were supplemented to a final concentration of 2.5 mM each. Anoxic SFU stocks of glyburide (Millipore Sigma, 356,310), glipizide (Millipore Sigma, G117-1G) and glimepiride (Millipore Sigma, G2295) were created at the indicated concentrations in DMSO and spiked into culture plates to achieve the final indicated SFU concentrations and DMSO percentages. The upper concentration for each SFU titration was initially selected based upon estimates for maximum in vivo concentrations assuming typical daily dosing and gut volume. These were later revised downward due to constraints arising from aqueous solubility and DMSO (vehicle) toxicity. Titrations were five-step two-fold dilution series from these upper concentrations. Culture plates were inoculated from frozen stocks of each strain and growth over 24-72 h was monitored via OD 600 nm, in an Epoch2 plate reader (Biotek Instruments, Inc). No-DMSO and no-SFU (DMSO-only) controls, as well as uninoculated controls, were included on each culture plate.

### Statistical analysis and graphics

As with all post hoc exploratory investigations, the methods and analyses presented here were not prescribed in the original study protocol nor Statistical Analysis Plan. Statistical significances are nevertheless provided as nominal ‘*p* values’ with comparison statistics, reflecting their value in description, disclosure, and ranking. All data analysis was performed using R version 4.0.2 [85]. All figures presented here can be reproduced exactly using the provided tidy [86] data files and R markdown [87] notebooks provided in a supplementary data compendium, hosted with other study data on GitHub at https://github.com/wholebiome/NCT03893422. Robust rank-based comparison (Wilcoxon Rank Sum [37]) of within- and between-group differences was performed using the ‘wilcox.text’ of the ‘stats’ package in R. One- or two-sample and sidedness of tests are indicated with their respective description in Results and figure captions.

For consistency with the previous description of results [21], the comparison of within- and between-group changes in post hoc re-analysis of glucose control endpoints uses a standard Student’s t-test (‘t.test’) as originally prescribed prior to unblinding in the 2018 statistical analysis plan for the study, NCT03893422. An exploratory multivariate summary of baseline or log-ratio metabolite change values used standard Principal Component Analysis (‘PCA’) [41] implemented in the FactoMineR package [88]. Robust regression [36] was performed using the ‘robustbase’ package [89]. The figures presented herein make extensive use of the ggplot2 package [90], with help from supporting packages ‘patchwork’ [91], ‘ggbeeswarm’ [92], ‘ggridges’ [93], and ‘ggrepel’ [94]. Data processing, tidying, and joining was accomplished with help from ‘magrittr’ [95] and ‘data.table’ [96] packages. Growth curve data was smoothed via polynomial spline implemented in the pspline package [97]. The conceptual overview in Fig. 1 was created using the BioRender.com web application. Supplementary reports corresponding to each data figure were created using the R markdown [87] and rmdformats [98] packages.

### Bacterial genomic analysis

Formulation strain genomes were sequenced using a PacBio Sequel and assembled using HiCanu [99]. To correct for sequencing errors in the PacBio assemblies, each genome was additionally sequenced using an Illumina Miseq sequencer and MiSeq Reagent Kit v3 (600-cycle, MS-102-3003) 2 × 300 bp paired-end mode. Library sizes were sufficient to achieve coverages of 500X, 250X, 250X and 200X for AMUC, EHAL, CBUT, and CBEI, respectively. The assemblies were corrected using Pilon [100] and completeness in terms of circular contigs was checked using Circlator [101]. The BINF genome was sequenced using PacBio RSII without additional short read sequencing. The query amino acid sequences of proteins involved in bile acid metabolism were obtained from Uniprot [102], limiting inclusion to Uniprot status ‘reviewed’. Candidate genes on formulation strain genomes were identified using Diamond [103] and e-value maximum threshold of 10^− 10^. Identification of an identical P9-encoding gene in strain AMUC (GenBank: CP073284, locus tag KDJ95_08250) was accomplished by mapping alignment [104] of the sequence of the P9 gene (locus tag: Amuc_1631) at nucleotide positions 1,965,361..1967607 of *Akkermansia muciniphila* ATCC BAA-835 (GenBank: CP001071), with graphical inspection facilitated by Geneious Prime 2020.2.5.

## Supplementary Information

**Additional file 1.**

## Data Availability

All study data and code required to exactly reproduce the analysis, tables, and figures described herein are included in supplementary files as well as the GitHub repository https://github.com/wholebiome/NCT03893422. Metabolite data has also been deposited at the MetaboLights [105] repository under study reference identifier MTBLS2713. The complete genome sequences of each of the five study probiotic strains were deposited with NCBI under BioProject PRJNA722306 and GenBank accession numbers CP073277 - CP073284.

## Abbreviations

AHAS: Acetohydroxyacid synthase (EC 2.2.1.6)
AMUC: *Akkermansia muciniphila* strain AMUC
BCAA: Branched chain amino acids
BINF: *Bifidobacterium infantis* strain BINF
CA: Cholic acid
CBEI: *Clostridium beijerinckii* strain CBEI
CBUT: *Clostridium butyricum* strain CBUT
(G)CDCA: (glyco-)chenodeoxycholic acid
DMSO: Dimethylsulfoxide
EHAL: *Anaerobutyricum hallii* strain EHAL
ESI: Electrospray ionization
FDR: False discovery rate
FXR: Farnesoid X receptor
GLP-1: Glucagon-like peptide 1
HbA1c: Standard measurement of glycated haemoglobin
MS/MS: Tandem mass spectrometry
PCA: Principal Component Analysis
PYG: Peptone, yeast extract, glucose medium
SCFA: Short-chain fatty acid
SFU: Sulfonylurea
T2D: Type 2 diabetes
TGR5: G protein-coupled bile acid receptor 1, also known as GPBAR1, GPCR19, M-BAR
(G)UDCA: (glyco)ursodeoxycholate
UHPLC: Ultra high performance liquid chromatography
WBF-010 & WBF-011: 3-strain and 5-strain probiotic formulations defined in Fig. 1, respectively
