# Supplementary material for: Increased circulating butyrate and ursodeoxycholate during probiotic intervention in humans with type 2 diabetes
Source: BMC Microbiol. 2022 Jan 8;22:19. doi: 10.1186/s12866-021-02415-8 (PMC8742391; doi:10.1186/s12866-021-02415-8)
Supplement: Supplementary file 1 — Additional file 1. [file 12866_2021_2415_MOESM1_ESM.docx]

#

| gene_name | Entry name | Uniprot status | Protein names |
| --- | --- | --- | --- |
| BaiG | BAIG_CLOSV | reviewed | Bile acid transporter |
| BaiB | BAIB_CLOSV | reviewed | Bile acid-coenzyme A ligase (EC 6.-.-.-) |
| BaiA | BAIA1_CLOSV | reviewed | 3alpha-hydroxy bile acid-CoA-ester 3-dehydrogenase 1/3 (EC 1.1.1.395) (3alpha-hydroxysteroid dehydrogenase 1/3) (3alpha-HSDH 1/3) (Bile acid-inducible protein BaiA1) (Bile acid-inducible protein BaiA3) |
| BaiA | BAIA2_CLOSV | reviewed | 3alpha-hydroxy bile acid-CoA-ester 3-dehydrogenase 2 (EC 1.1.1.395) (3alpha-hydroxysteroid dehydrogenase 2) (3alpha-HSDH 2) (Bile acid-inducible protein BaiA2) |
| BaiF | BAIF_CLOSV | reviewed | Bile acid-CoA transferase (EC 2.8.3.25) (Bile acid-coenzyme A transferase) (Bile acid-inducible operon protein F) |
| BaiCD | BAICD_CLOSV | reviewed | 3-oxocholoyl-CoA 4-desaturase (EC 1.3.1.115) (Bile acid-inducible operon protein C) (Bile acid-inducible operon protein CD) (Bile acid-inducible operon protein D) |
| BaiH | BAIH_CLOSV | reviewed | 7-beta-hydroxy-3-oxochol-24-oyl-CoA 4-desaturase (EC 1.3.1.116) (NADH-dependent flavin oxidoreductase) (NADH:flavin oxidoreductase) (NADH:FOR) |
| BaiI | BAII_CLOSV | reviewed | Bile acid-inducible operon protein I (Fragment) |
| BaiE | BAIE_CLOSV | reviewed | Bile acid 7-alpha dehydratase (BA7 alpha dehydratase) (EC 4.2.1.106) (Bile acid-inducible operon protein E) |
| BaiN | BAIN_CLOS5 | reviewed | 3-dehydro-bile acid delta(4,6)-reductase (EC 1.3.1.114) |
| BaiN | C8WPA1_EGGLE | unreviewed | HI0933 family protein |
| BaiO | B0NAQ3_CLOS5 | unreviewed | Uncharacterized protein |
| BaiO | C8WPA2_EGGLE | unreviewed | FAD dependent oxidoreductase |
| BaiP | B0NCT7_CLOS5 | unreviewed | Amino acid carrier protein |
| BaiP | C8WPA0_EGGLE | unreviewed | Amino acid carrier protein |
| 3aHSDH | 3AHD_EGGLE | reviewed | 3alpha-hydroxysteroid dehydrogenase (3alpha-HSDH) (EC 1.1.1.-) (3alpha-hydroxycholanate dehydrogenase (NAD(+))) (EC 1.1.1.52) (NAD-dependent bile acid 3alpha-dehydrogenase) |
| BaiA | 3AHDP_RUMGV | reviewed | 3alpha-hydroxysteroid dehydrogenase (3alpha-HSDH) (EC 1.1.1.-) (3alpha-hydroxycholanate dehydrogenase (NADP(+))) (EC 1.1.1.392) (NADP-dependent bile acid 3alpha-dehydrogenase) |
| BSH | CBH_CLOPE | reviewed | Conjugated bile acid hydrolase (CBAH) (EC 3.5.1.-) (Bile salt hydrolase) (BSH) (CBAH-1) (Choloylglycine hydrolase) (EC 3.5.1.24) |
| BSH | CBH_BIFLN | reviewed | Conjugated bile acid hydrolase (EC 3.5.1.-) (Bile salt hydrolase) (BSH) (Chenodeoxycholoyltaurine hydrolase) (EC 3.5.1.74) (Choloylglycine hydrolase) (EC 3.5.1.24) |
| 7bHSDH | HSDHB_RUMGN | reviewed | 7beta-hydroxysteroid dehydrogenase (7beta-HSDH) (EC 1.1.1.201) (NADP-dependent 7beta-hydroxysteroid dehydrogenase) |
| 7bHSDH | HSDHB_COLAA | reviewed | 7beta-hydroxysteroid dehydrogenase (7beta-HSDH) (EC 1.1.1.201) (NADP-dependent 7beta-hydroxysteroid dehydrogenase) |
| 7aHSDH | HDHA_PAESO | reviewed | NADP-dependent 7-alpha-hydroxysteroid dehydrogenase (7-alpha-HSDH) (EC 1.1.1.-) (Bile acid 7-dehydroxylase) |
| 7aHSDH | HDHA_ECOLI | reviewed | 7-alpha-hydroxysteroid dehydrogenase (7-alpha-HSDH) (EC 1.1.1.159) |

**Supplementary Table 1**: Summary of Uniprot references included in search of enzymes predicted to modify bile acids encoded in the genomes of study formulation strains.


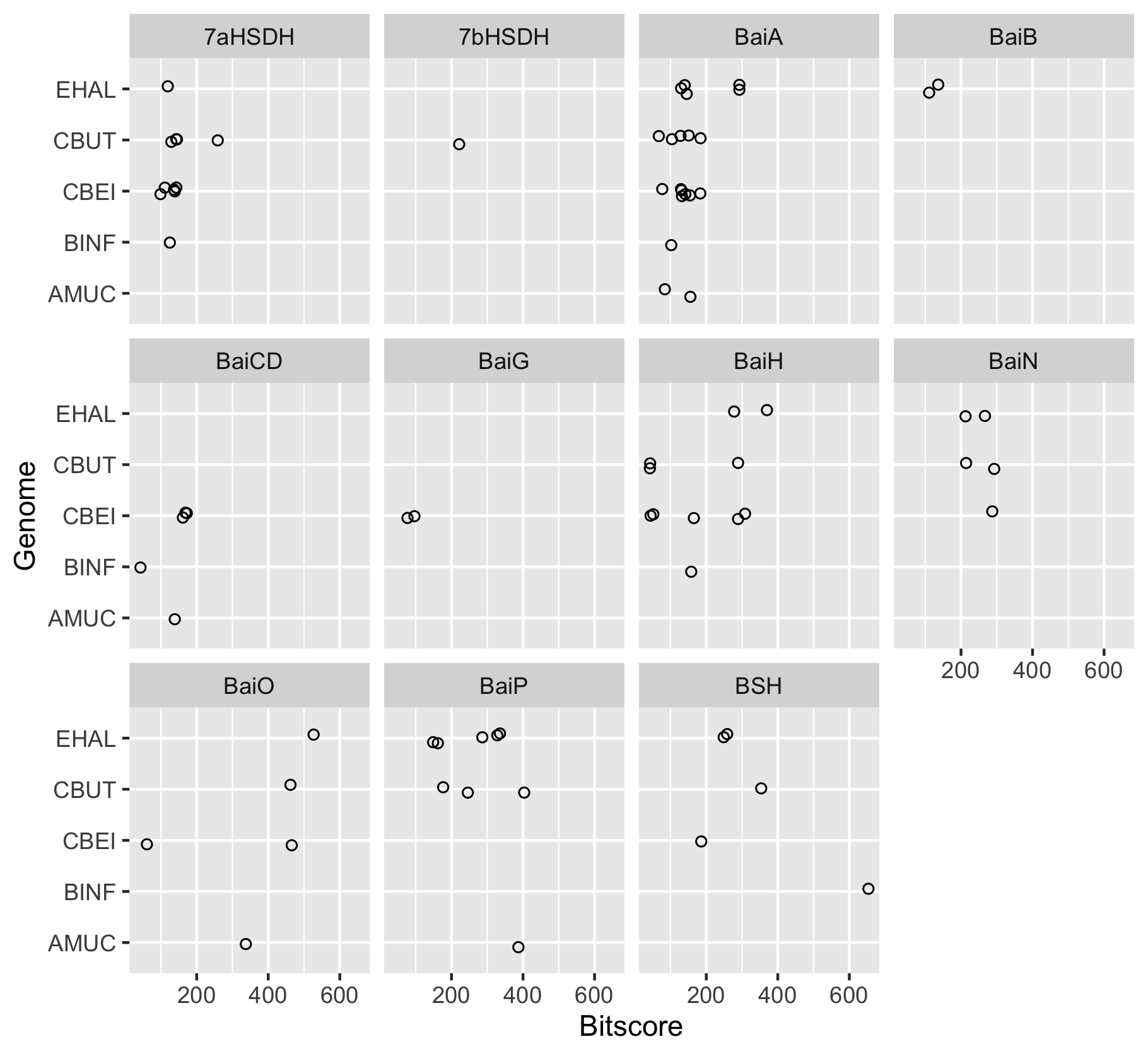


**Supplementary Figure 1**: Search results for bile acid modification genes in genomes of study formulation strains. The genes BaiE, BaiF, BaiI did not deliver any hits.

#

| Tube Number | Inoculated | Media | Time | Bile Acid Amendment | Starting Concentration [uM] | Sample Type |
| --- | --- | --- | --- | --- | --- | --- |
| 1 | FALSE | pyg | T0 | CDCA | 50 | supernatant |
| 2 | FALSE | pyg | T0 | CDCA | 50 | supernatant |
| 3 | FALSE | pyg | T0 | CDCA | 50 | supernatant |
| 4 | FALSE | pyg | T0 | CA | 50 | supernatant |
| 5 | FALSE | pyg | T0 | CA | 50 | supernatant |
| 6 | FALSE | pyg | T0 | CA | 50 | supernatant |
| 7 | TRUE | pyg | T0 | CA | 50 | supernatant |
| 8 | TRUE | pyg | T0 | CA | 50 | supernatant |
| 9 | TRUE | pyg | T0 | CA | 50 | supernatant |
| 10 | TRUE | pyg | T0 | CDCA | 50 | supernatant |
| 11 | TRUE | pyg | T0 | CDCA | 50 | supernatant |
| 12 | TRUE | pyg | T0 | CDCA | 50 | supernatant |
| 13 | FALSE | pyg | Tfinal | CDCA | 50 | supernatant |
| 14 | FALSE | pyg | Tfinal | CDCA | 50 | supernatant |
| 15 | FALSE | pyg | Tfinal | CDCA | 50 | supernatant |
| 16 | FALSE | pyg | Tfinal | CA | 50 | supernatant |
| 17 | FALSE | pyg | Tfinal | CA | 50 | supernatant |
| 18 | FALSE | pyg | Tfinal | CA | 50 | supernatant |
| 19 | TRUE | pyg | Tfinal | CA | 50 | supernatant |
| 20 | TRUE | pyg | Tfinal | CA | 50 | supernatant |
| 21 | TRUE | pyg | Tfinal | CA | 50 | supernatant |
| 22 | TRUE | pyg | Tfinal | CDCA | 50 | supernatant |
| 23 | TRUE | pyg | Tfinal | CDCA | 50 | supernatant |
| 24 | TRUE | pyg | Tfinal | CDCA | 50 | supernatant |
| 25 | TRUE | pyg | Tfinal | CA | 50 | cell pellet |
| 26 | TRUE | pyg | Tfinal | CA | 50 | cell pellet |
| 27 | TRUE | pyg | Tfinal | CA | 50 | cell pellet |
| 28 | TRUE | pyg | Tfinal | CDCA | 50 | cell pellet |
| 29 | TRUE | pyg | Tfinal | CDCA | 50 | cell pellet |
| 30 | TRUE | pyg | Tfinal | CDCA | 50 | cell pellet |

**Supplementary Table 2.** Experiment sampling design of *in vitro* monoculture for targeted measure of bile acids modification. Each row is a separate specimen collected at the indicated timepoint. Replicates are denoted by repeated row entries with the same values. Amendment of human primary bile acid indicated by their abbreviation. Cells are inoculated into sterile rich media amended with 50 uM final concentration of either cholic acid (CA) or chenodeoxycholic acid (CDCA).
